# Is COVID‐19 severity associated with reduction in T lymphocytes in anti‐CD20‐treated people with Multiple Sclerosis or Neuromyelitis Optica Spectrum Disorder?

**DOI:** 10.1111/cns.13841

**Published:** 2022-04-12

**Authors:** Robert Hoepner, Christian P Kamm, Christoph Friedli, Anke Salmen, Andrew Chan

**Affiliations:** ^1^ Department of Neurology Inselspital Bern University Hospital and University of Bern Bern Switzerland; ^2^ Neurocentre Cantonal Hospital Lucerne Lucerne Switzerland

## CONFLICT OF INTEREST

Dr Hoepner received speaker/advisor honorary from Merck, Novartis, Roche, Biogen, Alexion, Sanofi, Janssen, Bristol‐Myers Squibb, and Almirall. He received research support within the last 5 years from Roche, Merck, Sanofi, Biogen, Chiesi, and Bristol‐Myers Squibb. He also received research grants from the Swiss MS Society. He also serves as associated editor for Journal of Central Nervous System disease. All are not related to that work. Dr Kamm has received honoraria for lectures and research support from Biogen, Novartis, Almirall, Teva, Merck, Sanofi Genzyme, Roche, Eli Lilly, Janssen, Celgene, and the Swiss MS Society (SMSG). Friedli C received speaker honoraria and/or travel compensation for activities with Biogen, Sanofi Genzyme, Novartis, and Merck and research support from Chiesi, not related to this work. Dr Salmen received speaker honoraria and/or travel compensation for activities with Bristol‐Myers Squibb, Novartis, Roche, and research support by Baasch Medicus Foundation and the Swiss MS Society. She serves on the Editorial Board of Frontiers in Neurology—Multiple Sclerosis and Neuroimmunology. All are not related to this work. Dr Chan has received speakers’/board honoraria from Actelion (Janssen/J&J), Almirall, Bayer, Biogen, Celgene (BMS), Genzyme, Merck KGaA (Darmstadt, Germany), Novartis, Roche, and Teva, all for hospital research funds. He received research support from Biogen, Genzyme, and UCB, the European Union, and the Swiss National Foundation. He serves as associate editor of the European Journal of Neurology, on the editorial board for Clinical and Translational Neuroscience and as topic editor for the Journal of International Medical Research.

## DATA AVAILABILITY STATEMENT

Data can be obtained via corresponding author.

Corona Virus Disease (COVID)‐19 has a more severe course in people treated with anti‐CD20 therapies.^1^ In addition to depletion of B cells with reduced antibody responses,^2^, ^3^ a decrease in CD3^+^ T lymphocytes, especially CD8^+^ cytotoxic T cells,^4^ by anti‐CD20 drugs may be causative as particularly CD8^+^ T cell responses are discussed to be associated with COVID‐19 severity.^2^ The aim of our letter was to investigate whether T cell counts (CD3^+^, CD4^+^, or CD8^+^) are different in anti‐CD20‐treated patients (Ocrevus®, Roche 10/14; MabThera®, Roche 4/14) with severe (hospitalized, *n* = 7) or mild (outpatient managed, *n* = 7) COVID‐19 course.

In total, 14 patients with multiple sclerosis (MS, 11/14) or neuromyelitis optica spectrum disorder (NMOSD 3/14) receiving anti‐CD20 drugs, who had a severe acute respiratory syndrome coronavirus type 2 (SARS‐CoV2) infection while on anti‐CD20 treatment, were identified by medical chart review (Cantonal Hospital Lucerne: 5/14, ethic vote: 2020–00044; University Hospital Bern 9/14, NI registry ethic vote: 2017–01369, last amendment August 2020). COVID‐19 was diagnosed by polymerase chain reaction (PCR) test between March 2020 and July 2021 in all cases.

Patients were, in majority, female (9/14), 45.5 years old (median, 25th–75th percentile (25th–75th) 28.8–52.3, *n* = 14), and had an expanded disability status scale (EDSS) score of 2.0 (median, 25th–75th 0–3.55, *n* = 14). Time between last infusion and COVID‐19 infection was 4.4 months (median, 25th–75th 1.8–5.7, *n* = 14). All but one anti‐CD20‐treated patients with COVID‐19 were not vaccinated prior COVID‐19. The single patient vaccinated 2 months prior infection (Spikevax®, Moderna) was treated with Ocrevus® and managed on an outpatient basis. Further cohort characteristics are displayed in the figure. Out of 14, 7 patients were hospitalized due to COVID‐19 (4/7 MS, 3/7 NMOSD) of whom 2/7 were treated in the intensive care unit and 1/2 required artificial ventilation. All patients recovered without sequelae.

**FIGURE 1:**
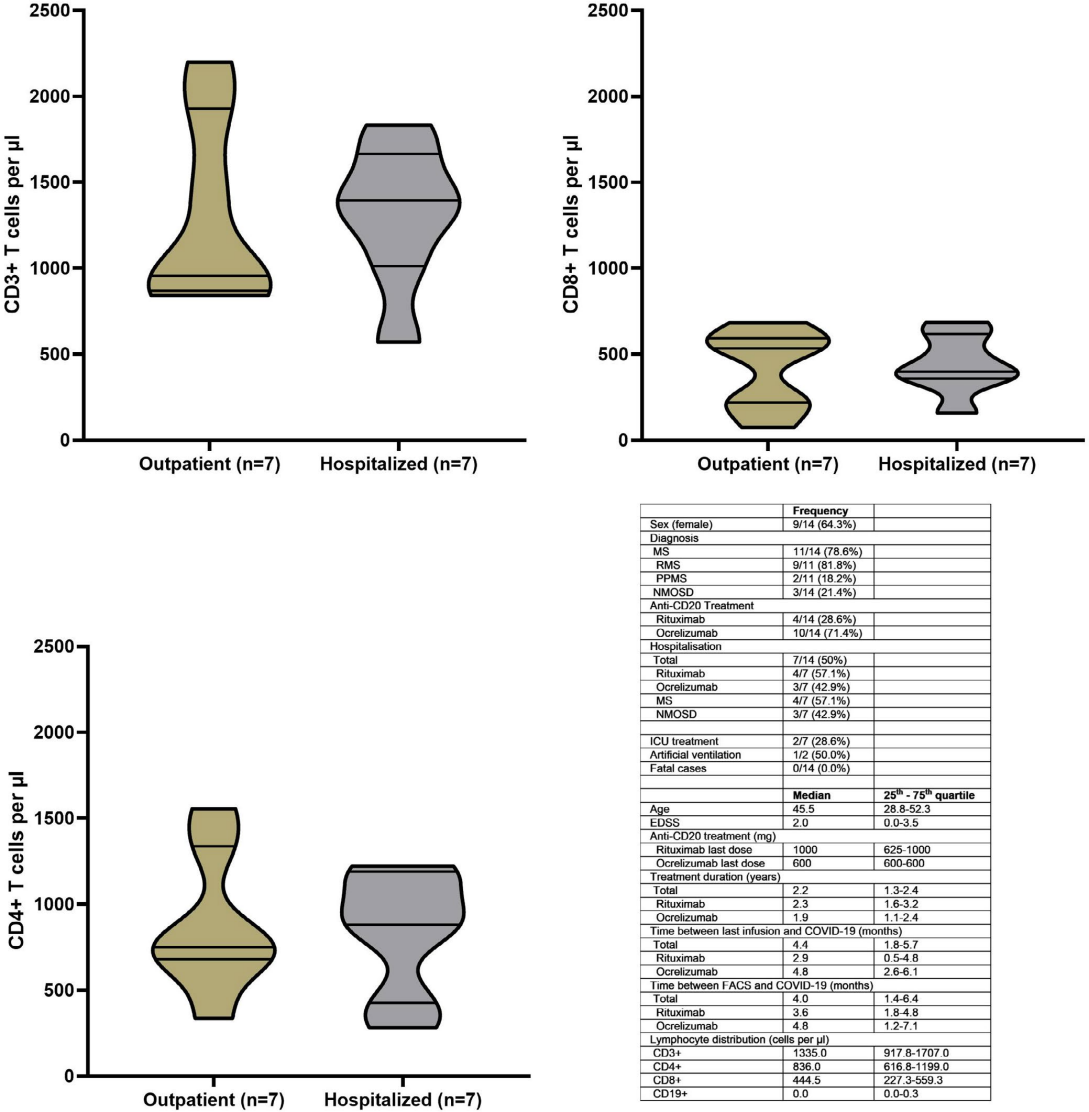
Table presenting the cohort characteristics and violin plots demonstrating the distribution of CD3^+^, CD8^+^, and CD4^+^ lymphocytes between outpatient managed (bronze) and hospitalized (gray) patients with COVID‐19 while on anti‐CD20 treatment. Violin plots display median, 25th and 75th quartiles and minimum/maximum. Mann–Whitney test demonstrated no significant differences with p‐values as follows: CD3^+^ *p* = 0.90, CD4^+^ *p* = 0.90, CD8^+^ *p* = 0.90, and CD19^+^ *p* = 0.73 (CD19^+^ not displayed as violin plot). The figure was created with GraphPad Prism Version 8.0.1. Abbreviations: COVID‐19, Corona Virus Disease‐19, EDSS, Expanded Disability Status Scale, FACS, fluorescence‐activated cell sorting, ICU, intensive care unit, MS, multiple sclerosis, NMOSD, neuromyelitis optica spectrum disorder, PPMS, primary progressive MS, RMS, relapsing MS

Diagnosis (MS vs NMOSD), age, sex, anti‐CD20 drug used, last administered anti‐CD20 dose (milligram), EDSS, time since last infusion (months), and overall treatment duration (years) did not differ between hospitalized and non‐hospitalized patients (Mann–Whitney or Fisher's exact test, all *p* > 0.05). Fluorescence‐activated cell sorting (FACS) analysis was performed 4.0 months prior COVID‐19 (median, 25th–75th 1.4–6.4). B cell populations were depleted in 11/14, and the remaining three patients had a B cell count of 1/µl, 2/µl, or 55/µl, respectively. Median number of T cell (CD3^+^, CD8^+^, and CD4^+^) counts is displayed in the figure without differences between hospitalized and outpatient managed COVID‐19 cases (Figure 1).

Our small case series did not demonstrate differences in T lymphocyte populations between hospitalized and non‐hospitalized patients with COVID‐19 during anti‐CD20 treatment. Concerning previous studies, a database research run until July 31, 2020, identified 51 COVID‐19 cases out of 4000 patients included in ongoing Roche/Genentech clinical trials. Despite the majority of patients (35/51) having a mild‐to‐moderate disease course, 16/51 were hospitalized, 10/51 had a severe disease course, and 3/51 died.^5^ Despite small patient numbers, 3/51 exceeds the worldwide COVID‐19 fatality rates (1.5%^6^; MS specific 3.3% (95%‐confidence interval (95%CI), 2.5%–4.3%^7^). Increased COVID‐19 severity was also demonstrated by an analysis of a more homogenous cohort of MS patients treated in France and Italy^8^ demonstrating an odds ratio (OR) of 2.05 (95%CI 1.39–3.02). In contrast to the previous article, the highest grade of severity was not only restricted to death but also included hospitalization in an intensive care unit (ICU), which might be prone to bias concerning physicians having a lower threshold for an ICU hospitalization in ocrelizumab‐treated MS patients. Nevertheless, a study of our group, focusing on patients with any autoimmune disease treated with anti‐CD20 medications and being listed in the FDA Adverse Event Reporting System, confirmed a higher risk of death due to COVID‐19 in anti‐CD20‐treated patients (OR 4.5 95%CI 2.6–7.9) even after adjustment for the local dynamics of the pandemic and the performance of the national health care system.^1^ However, despite the known patient‐related risk factors such as age and ambulatory disability,^7^ anti‐CD20 treatment‐related risk factors such as reduction in T cell counts are still unknown justifying our small case series and hopefully stimulating further research in this field. One reason for negative results of our case series might be that the overall severity of COVID‐19 was still relatively moderate without any death related to COVID‐19. The main limitation of our article is the small sample size, which might be too small to detect statistical differences. We, therefore, view our data as hypothesis‐generating and ask other researchers to share their anonymized data with our research group (contact for data sharing with us or requesting our data via corresponding authors) in order to overcome this limitation.

## References

[1] Pistor M , Hoepner AGF , Lin Y , et al. Immunotherapies and COVID‐19 mortality: a multidisciplinary open data analysis based on FDA's Adverse Event Reporting System. Ann Rheum Dis. 2021;80(12):1633‐1635. 34285050 10.1136/annrheumdis-2021-220679 PMC8600608

[2] Poland GA , Ovsyannikova IG , Kennedy RB . SARS‐CoV‐2 immunity: review and applications to phase 3 vaccine candidates. Lancet. 2020;396(10262):1595‐1606. 33065034 10.1016/S0140-6736(20)32137-1 PMC7553736

[3] Furlan A , Forner G , Cipriani L , et al. COVID‐19 in B cell‐depleted patients after rituximab: a diagnostic and therapeutic challenge. Front Immunol. 2021;12:763412. 34804051 10.3389/fimmu.2021.763412 PMC8595333

[4] Gingele S , Skripuletz T , Jacobs R . Role of CD20(+) T cells in multiple sclerosis: implications for treatment with ocrelizumab. Neural Regen Res. 2020;15(4):663‐664. 31638088 10.4103/1673-5374.266913 PMC6975146

[5] Hughes R , Whitley L , Fitovski K , et al. COVID‐19 in ocrelizumab‐treated people with multiple sclerosis. Mult Scler Relat Disord. 2021;49:102725. 33482590 10.1016/j.msard.2020.102725 PMC7772086

[6] Anonymos . 2022. Available from: https://www.worldometers.info/coronavirus/

[7] Salter A , Fox RJ , Newsome SD , et al. Outcomes and Risk Factors Associated With SARS‐CoV‐2 Infection in a North American Registry of Patients With Multiple Sclerosis. JAMA Neurol. 2021;78(6):699‐708. 33739362 10.1001/jamaneurol.2021.0688 PMC7980147

[8] Sormani MP , Salvetti M , Labauge P , et al. DMTs and Covid‐19 severity in MS: a pooled analysis from Italy and France. Ann Clin Transl Neurol. 2021;8(8):1738‐1744. 34240579 10.1002/acn3.51408 PMC8351392

